# Epithelioid Sarcoma—From Genetics to Clinical Practice

**DOI:** 10.3390/cancers12082112

**Published:** 2020-07-29

**Authors:** Anna M. Czarnecka, Pawel Sobczuk, Michal Kostrzanowski, Mateusz Spalek, Marzanna Chojnacka, Anna Szumera-Cieckiewicz, Piotr Rutkowski

**Affiliations:** 1 Department of Soft Tissue/Bone Sarcoma and Melanoma, Maria Sklodowska-Curie National Research Institute of Oncology, 02-781 Warsaw, Poland; mkostrzan@gmail.com (M.K.); mateusz@spalek.co (M.S.); piotr.rutkowski@pib-nio.pl (P.R.); 2 Department of Experimental Pharmacology, Mossakowski Medical Research Centre, Polish Academy of Sciences, 02-106 Warsaw, Poland; 3 Department of Experimental and Clinical Physiology, Laboratory of Centre for Preclinical Research, Medical University of Warsaw, 02-097 Warsaw, Poland; 4 Faculty of Medicine, Medical University of Warsaw, 02-091 Warsaw, Poland; 5 Department of Radiotherapy, Maria Sklodowska-Curie National Research Institute of Oncology, 02-781 Warsaw, Poland; marzanna.chojnacka@pib-nio.pl; 6 Department of Pathology and Laboratory Diagnostics, Maria Sklodowska-Curie National Research Institute of Oncology, 02-781 Warsaw, Poland; anna.szumera-cieckiewicz@pib-nio.pl; 7 Department of Diagnostic Hematology, Institute of Hematology and Transfusion Medicine, 02-776 Warsaw, Poland

**Keywords:** epithelioid sarcoma, SMARCB1, EZH2, surgery, radiotherapy, chemotherapy, tazemetostat

## Abstract

Epithelioid sarcoma is a mesenchymal soft tissue sarcoma often arising in the extremities, usually in young adults with a pick of incidence at 35 years of age. Epithelioid sarcoma (ES) is characterized by the loss of SMARCB1/INI1 (integrase interactor 1) or other proteins of the SWI/SNF complex. Two distinct types, proximal and distal, with varying biology and treatment outcomes, are distinguished. ES is known for aggressive behavior, including a high recurrence rate and regional lymph node metastases. An optimal long-term management strategy is still to be defined. The best treatment of localized ES is wide surgical resection. Neo-adjuvant or adjuvant radiotherapy may be recommended, as it reduces the local recurrence rate. Sentinel lymph node biopsy should be considered in ES patients. Patients with metastatic ES have a poor prognosis with an expected median overall survival of about a year. Doxorubicin-based regimens are recommended for advanced ES. Tazemetostat, an EZH2 methyltransferase, has shown promising results in ES patients. Novel therapies, including immunotherapy, are still needed.

## 1. Introduction

Epithelioid sarcoma (ES) is a rare, slow-growing neoplasm that was first well-characterized by F.M. Enzinger in 1970 [1]. ES is a soft tissue sarcomas (STS) subtype that is recognized in less than 1% of STS patients [2]. However, it is the second most common STS of the hand and the sixth STS of the upper extremity [2,3]. In a study of 16,500 subsequent STS patients, only 23 ES were reported, but it was the most common STS: of the hand [4]. The ES incidence rate is 0.03/100,000 in the European Union and 0.05/10,000 in the USA as per RARECAREnet and the SEER18 cancer registries. Age-adjusted rate in EU and USA is 0.02/100,000 and 0.05/100,000, respectively [5]. ES usually develops in young to middle-aged adults (20–40 years of age group) [6,7]. Distal subtype occurs mostly in young adults, while proximal is more common in a slightly older population, with a median age of 40 years at the time of diagnosis [6,8]. ES is very unlikely to occur in children; however, a series of cases were reported, as described below [9,10,11]. Males suffer from ES more often than females, with ratio of 2:1 [2,12,13].

**Figure 1:**
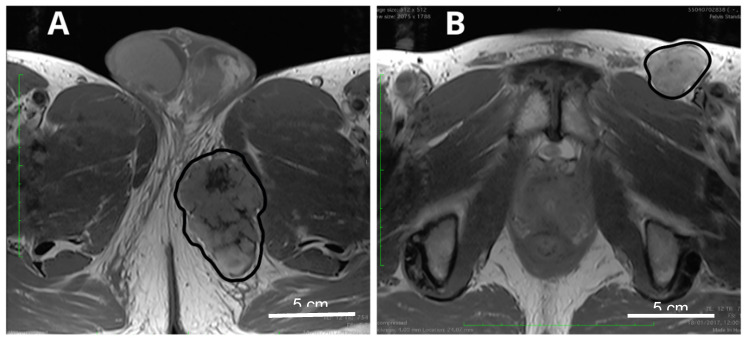
Epithelioid sarcoma—perineal area. (**A**) Locally advanced primary tumor (delineated, black contour); (**B**) Metastases to regional lymph nodes in the same patient (delineated, black contour).

Two ES subtypes—distal and proximal—are known. These subtypes vary in morphology and prognosis but occur at a similar rate. The distal ES subtype is the canonical subtype of the disease, and it most often presents as a deep dermal or subcutaneous tumor with lymphatic node metastases. Proximal ES develops mostly in the proximal limbs, pelvis, perineum, and genital tract [14] (Figure 1A). Each of the subtypes develops both in proximal and distal locations [6,15]. In general, the most common ES localization is in the extremities. Still, few cases with atypical ES localization in small bowel, penis, vulva, tongue, buttocks, parotid gland, palate, or intraocular are known [16,17,18]. Around a quarter of cases occur in the location previously affected by trauma or in the scar tissues [19].

At presentation, the disease typically manifests as a painless, slow-growing, firm nodule deep in soft tissues with a glistening and gray-tan appearance characterized by superficial bleeding, necrosis, and ulcerations [14]. Pain is reported by some patients, mainly if tumors localize in proximity to joints. Various morphology, symptoms, and signs lead to difficulties in ES diagnostics and often delay appropriate treatment. Due to the ulceration differential, diagnosis should include non-healing wounds and warts [20]. ES is also often initially recognized as inflammatory or granulomatous lesions or other benign conditions [21]. At the time of diagnosis, ES tumors are usually small with a diameter below 5 cm [13,22]; however, in some cases, mostly in proximal variant, ES tumors reach over 20 cm dimension [1,23]. In 10–20%, ES is multifocal. Tumors with deep tissue location spread via tendon sheaths and aponeuroses [13,19,24]. ES often metastasize to lymph nodes (up to 30%). Patients suffer from in-transit metastases and lymphatic spread (Figure 1B). [22,24]. Distant metastases most often develop in the lungs, bones, and brain. Less common metastases are found in the scalp, kidneys, musculoskeletal system, and digestive tract, including the liver [25]. ES metastatic spread is reported in the course of the disease in 20% to 50% of patients [19,22,26]. Around 20% of patients present with distant metastases already at the primary diagnosis [27].

## 2. Pathology

**Figure 2:**
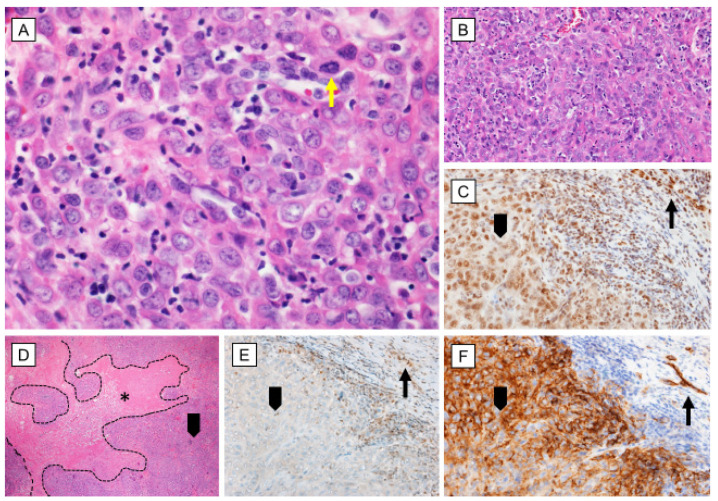
Epithelioid sarcoma—morphology and immune profile. (**A**), (**B**), (**D**) Epitheliod, large cells with abundant eosinophilic cytoplasm and moderate nuclear pleomorphism, mostly arranged in a solid sheath of neoplastic cells with marked geographical necrosis [hematoxylin-eosin 400×, 200× and 40× respectively]; (**C**) Positive strong nuclear reaction with FLI1 [200×]; (**E**) Immunohistochemical loss of INI–1 [200×]; (**F**) Positive strong reaction with CD34 [200×]; arrowhead—tumor cells; yellow arrow—mitotic figure; black arrow—internal control in endothelial cells; asterisk—necrosis.

**Figure 3:**
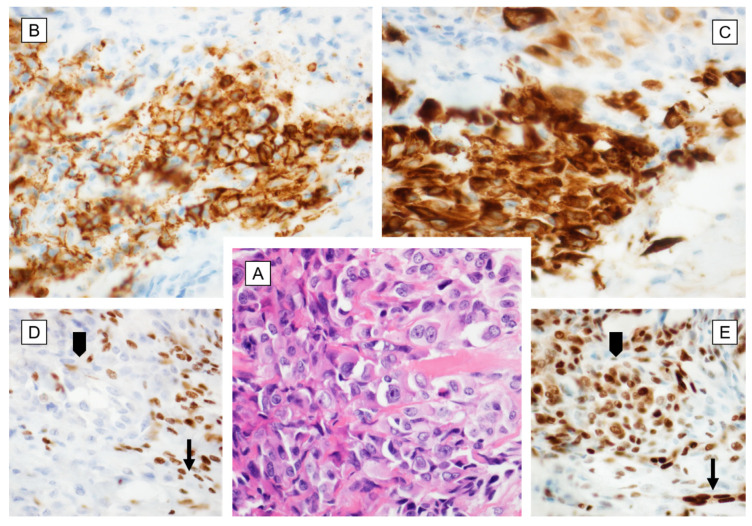
Epithelioid sarcoma—morphology and immune profile. (**A**) Epitheliod, large cells with abundant eosinophilic cytoplasm and moderate nuclear pleomorphism [HE, 200×]; (**B**) Positive strong reaction with CD34 [200×]; (**C**) Positive, focally strong reaction with cytokeratin AE1/AE3 (CKAE1/AE3) [200×]; (**D**) Immunohistochemical loss of integrase interactor 1 (INI1) [200×]; (**E**) Positive strong nuclear reaction with ERG [200×]; arrowhead—tumor cells; arrow—internal control in endothelial cells.

Microscopically ES is typically multinodular, often with a tendency to central necrosis and degeneration. With the hematoxylin–eosin staining, the deep acidophilia is observed. The nodular pattern could be variable—in some cases, the nodules are well-circumscribed, while in others, they can be fused into irregular lobulated masses. Cells vary from plum spindle to large round or polygonal (Figure 2A,B, and Figure 3A). Although the spindle cells predominate in early lesions, metastatic tumors are characterized by atypic, polygonal, and epithelioid cells. Mitoses are noted only occasionally [19,28,29]. Even though both proximal and distant variants belong to SWI/SNF-deficient soft tissue, neoplasms are unified by the presence of monomorphic, undifferentiated epithelioid cells and are characterized by the loss of INI1 expression (Figure 2E). ES has relatively heterogeneous morphology. Classical ES is characterized by comparatively unvarying, bland-looking epithelial cells with central necrosis, while the proximal type is composed of multinodular sheets of large polygonal cells with prominent nucleoli, sometimes with rhabdoid features and more prominent nuclear atypia [6,8]. Palisaded hyalinizing necrosis is a characteristic feature of the classical ES; usually, it is most prominent in the central zone, resulting in a pseudogranulomatous appearance (Figure 2D). Thus, the misclassification as a benign granulomatous or reactive process is frequent. Additionally, lymphocytic infiltration, hemorrhage, the deposition of hemosiderin, fibrin, or mucin are also observed. Occasionally, stromal changes such as focal calcification, desmoplasia, metaplastic ossification, or rarely myxoid changes can be found as well [13,19].

Immunohistochemically, ES cells present the features of mesenchymal and epithelioid cells. ES cells express vimentin, cytokeratins, epithelial membrane antigen (EMA), and variably ETS-transcription factors (ERG). Among the cytokeratins, the most commonly expressed are CK8 (>90%), CK19 (> 70%), and CK14 (50%) [28]. About 50% of tumors express CD34 and CA125, whereas the expression of smooth muscle actin is variable [14,26,28,30,31,32] (Figure 2F, Figure 3B). CA125 is also secreted into the blood by ES cells and could be utilized as a marker for the diagnosis and management of the disease [33]. Immunoreactivity for cytokeratin is cytoplasmatic, whereas EMA is observed exclusively on cell membranes [34]. ES cells are usually positive for ERG, Friend leukemia integration 1 transcription facto (FLI1), and cytokeratin AE1/AE3 (CKAE1/AE3), and they are negative for S100, desmin, and factor VIII [28,35,36] (Figure 2C, Figure 3E,C). Over 90% of ES, both conventional and proximal type, shows a complete SMARC1/INI1 (integrase interactor 1) loss due to the biallelic deletion of the *SMARCB1* gene locus or as a consequence of epigenetic dysregulation, which defines both types of epithelioid sarcoma [37]. In the appropriate context, immunohistochemistry for INI1 should be used to confirm the diagnosis (Figure 3D). However, in a few patients (especially in older groups or at the abnormal anatomic site), INI1 expression is retained [37]; thus, the diagnosis of ES should always be made with precaution.

ES may mimic various malignant lesions such as a malignant rhabdoid tumor, epithelioid malignant peripheral nerve sheath tumor, non-keratinizing skin carcinoma, epithelioid hemangioendothelioma, synovial sarcoma, anaplastic carcinoma, melanoma, anaplastic lymphoma, and even metastatic signet ring cell adenocarcinoma. Many types of carcinomas can have a similar presentation to ES but usually, the specific immunohistochemical profile enables diagnosis [34]. Hemorrhage into clefts can mislead the diagnosis toward epithelioid angiosarcoma or hemangioendothelioma [1]. The malignancies mentioned above are usually positive for endothelial markers such as CD31 and factor VIII, whereas ES in negative for those markers [19,28]. In the differential diagnosis, benign conditions such as giant cell tumors of the tendon sheath, benign fibrous histiocytoma, granuloma annulare, or necrotizing granulomas should be considered [38].

## 3. Genetics and Molecular Biology

Loss of integrase interactor 1 (INI1) function is the most common alteration found in ES, occurring in nearly 90% of cases [37]. INI1 is coded by the *SMARCB1* gene located on the long arm of chromosome 22 (22q11.2) and is a member of the SWI/SNF chromatin-remodeling complex. This complex mobilizes nucleosomes and causes the exposition of DNA to transcription factors. Biallelic inactivation of INI1 occurs in strictly defined malignant rhabdoid tumors of infancy, which confirmed its functions as a tumor-suppressor gene. *SMARCB1*/INI1 inactivation leads to the deregulation of targeted genes, uncontrolled cellular growth, and neoplastic transformation [39]. The restoration of *SMARCB1* resulted in the reduced cell proliferation and migration of the ES VAESBJ cell line [39]. INI1 function can be lost due to the homozygous deletion of *SMARCB1*, bi- or single-allelic deletion, or point mutations; however, in up to 50% of ES cases, both alleles of the gene are intact [40,41], which has led to the discovery that INI1 loss is caused rather at the epigenetic level or interaction with microRNA in the silencing of this gene [42].

Other mechanisms that play a crucial role in the pathogenesis of ES are the methylation events catalyzed by histone methyltransferases (HMTs) [43]. EZH2, EED, SUZ12, and RBAP48 are the subunits of the HMT complex known as polycomb repressive complex 2 (PRC2), which catalyzes the mono-, di-, and trimethylation of histone H3 lysine 27 (H3K27) [44]. The trimethylated form of H3K27Me3 is connected with the repression of genes crucial for cell differentiation. PRC2 activity is antagonized by the above-mentioned SWI/SNF complex. INI1 inactivation results in overactivation of the PRC2 complex, leading to the methylation of histones, promotion of cell proliferation, and silencing the genes responsible for differentiation [45]. It has been confirmed in experimental studies where the blockade of EZH2 could induce apoptosis and significantly retard the growth of INI1-negative solid tumors [46].

Those observations have opened the field for preclinical studies evaluating histone deacetylase inhibitors (HDACi) [45,47]. Histone deacetylases are the group of enzymes that deacetylase lysine on histone and non-histone proteins inducing changes in chromatin structure and, in the appropriate context, facilitates transcription of the genes involved in cell proliferation, differentiation, and apoptosis [48,49]. Treatment with HDACi inhibited colony formation, induced cell cycle arrest in the G2 phase, and induced apoptosis in the ES cell lines. Moreover, an abrogation of cell growth was both observed in in vitro and in vivo studies [47]. Based on those results, further clinical trials with HDACi tazemetostat were initiated [50].

Various other alterations have been identified in the ES, including activation of the phosphatidylinositol 3-kinase/protein kinase–B/mammalian target of rapamycin (PI3K/AKT/mTOR) signaling pathway [51,52,53] and overexpression of epidermal growth factor receptor (EFGR) [51,54]. Other studies showed that the mesenchymal-epithelial transition factor (c-MET) pathway is involved in ES growth. Imura et al. [53] published results showing that adding a selective c-MET inhibitor to the mTOR inhibitor leads to a stronger inhibition of ES xenograft growth than either agent alone. In addition, EGFR inhibitors showed preclinical efficacy alone and in combination with mTOR inhibitors, as shown by in vivo and in vitro models of ES growth [51].

High expression of the vascular endothelial growth factor (VEGF) is responsible for angiogenesis [55] and has been described in epithelioid sarcoma samples [56], which have found implication in the clinical utility of tyrosine kinase inhibitors, such as pazopanib; however, their efficacy is limited and is described in a further section. 

The dysregulation of adhesion proteins is also described in ES. Complete loss of E-cadherin, a glycoprotein responsible for cell–cell adhesion, has been reported [57]. It plays an important role in epithelial-to-mesenchymal transition; thus, its loss may be involved in the spread of the disease and development of metastases [58]. The loss of E-cadherin can be caused by the overexpression of dysadherin, which is a glycoprotein that acts as a negative regulator of E-cadherin [59]. Its higher expression was observed mainly in cells derived from proximal-type ES which can, to some extent, explain its worse prognosis [60].

Transcriptomic analyses of ES samples have revealed differences in the expression of various regulatory pathways between proximal and distal types [61]. Proximal ES presented with the overexpression of *MYC* and signatures impacting the cell cycle, protein synthesis, and chromatin metabolism. The distal variant is characterized by an enrichment in Notch/Hedgehog and immune system regulation pathways that predict increased class 1human leukocyte antigens (HLA) expression and more pronounced immune infiltration [61]. These observations may partially explain differences in response to current therapies and suggest possible future directions for research and clinical trials. 

A variety of other molecular and genetic abnormalities such as the loss of *PBRM1* and *SYT–SSX1* expression, overexpression of interleukin-2β, or *NRAS* mutation were reported in ES; their clinical significance has not been yet established [62]. Moreover, several ES cases with chromosomal abnormalities in the 22q region were reported [63]. Currently, there are no specific cytogenetic abnormalities that are pathognomonic for ES. Further analyses of the molecular biology and genetics of ES are needed. Preliminary characteristics of cancer stem-like cells/cancer-initiating cells of ES are available. The subpopulation of ES cells with the higher ALDH1 activity was characterized by a higher expression of CD109. The authors using a new human ES cell line connected the overexpression of CD109 with higher tumorigenicity in vivo, enhanced invasiveness in vitro*,* and clonogenic ability. CD109 is considered a potential ES cancer stem cell marker and target for ES therapies [64]. Moreover, it was also found in myxofibrosarcoma samples, which suggests its wider applicability in the soft tissue sarcomas [65] 

Due to the rarity of ES, its biology is still poorly understood, and further extensive research is necessary. With recent advantages in the area of patient-derived primary cultures and xenografts, organoids, mice, and zebrafish models, the natural history of the disease can be better characterized [66].

## 4. Surgical Treatment

### 4.1. Surgical Treatment of Localized Disease

The treatment of choice of ES is a radical excision with microscopically radical margins and perioperative radiotherapy after careful assessment in a multidisciplinary team. The role of radiotherapy (RT) is described in the next section. Isolated limb perfusion and flap reconstructions may be utilized. As a result of the common location in the distal part of extremities in cases with extensive soft tissue infiltration beyond the possibility of reconstruction with acceptable functional results, the amputation is sometimes necessary [24,67,68]. This distal localization with indolent growth may also lead to improper primary unplanned surgical procedures before referral to reference sarcoma center due to unexpected clinical presentation [67]. Moreover, the clinical course of this sarcoma subtype is characterized by a high risk of multiple recurrences, including multifocal in-transit metastases due to the spread of tumor cells along the fascia and tendon [12,23,24,25,29,67,68,69,70]. The 5-year risk of recurrence can even exceed 70% [12,15,24,70,71,72,73]. Positive surgical margins (R1 and R2 resections) were associated with a higher risk of recurrence [27]; however, some studies have not confirmed this observation [70,74].

### 4.2. Lymphadenectomy

As compared to other types of soft tissue sarcomas, the spread to regional lymph nodes can occur more frequently (> 20%) [75,76,77,78], but the most common site of metastasis is the lung and pleura [12,23,25,29,68,70,78]. This higher rate of nodal involvement may justify performing sentinel node biopsy in selected cases, but existing data show the low percentage of finding metastatic tumors in clinically noninvolved nodes [76,79,80,81]. In the case of the presence of lymph node metastasis, therapeutic lymph node dissection is indicated [23,67,78].

## 5. Radiotherapy 

### 5.1. Radiotherapy of the Primary Tumor

**Figure 4:**
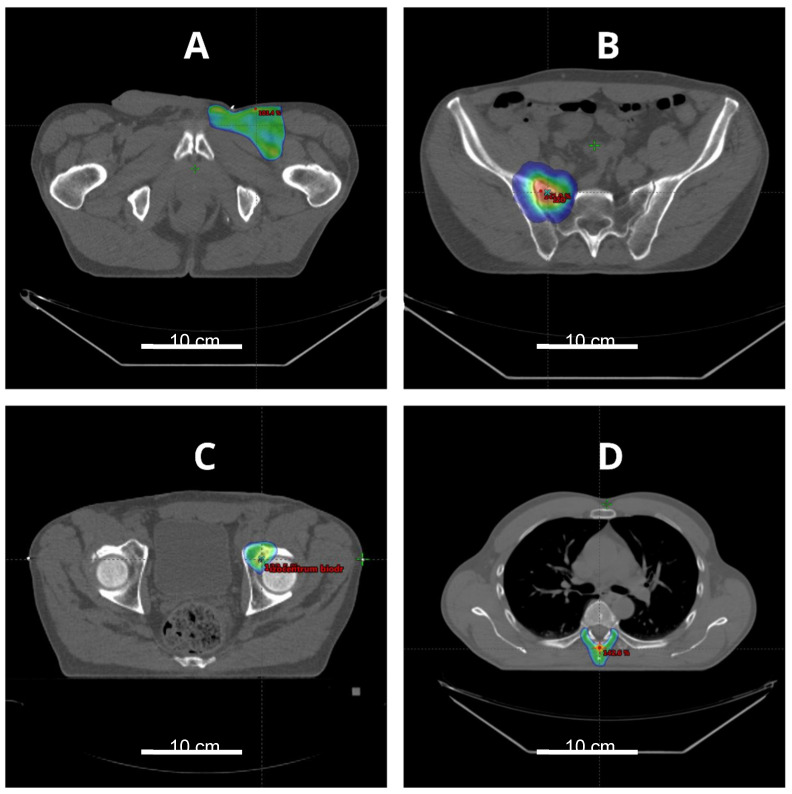
Radiotherapy plans in a patient with epithelioid sarcoma. (**A**) Adjuvant radiotherapy after resection of the primary tumor; (**B**–**D**) Stereotactic body radiotherapy for oligometastases in the same patient.

ES is generally considered radioresistant, even in comparison to the rest of soft tissue sarcomas. However, data related to definitive or perioperative RT in ES are scarce. However, RT is routinely used as an adjuvant to surgery (Figure 4A). The largest analysis found in the literature described 24 patients with nonmetastatic ES treated with perioperative RT and surgery [82]. Among them, only three patients received neoadjuvant RT with a median total dose of 46.4 Gy. The rest were irradiated postoperatively, with a median total dose of 64.5 Gy. Disappointingly, local failure occurred in 7 (29%) patients, which translated into a 10-year local control rate of 63%. In comparison to other soft tissue sarcomas with a local control rate of over 80%, that result seems to be unsatisfactory [83]. Another study published by Wolf et al. confirmed these findings, showing that among seven patients with primary ES who underwent adjuvant RT, 5 (71%) developed local recurrence [84]. Moreover, neither adjuvant RT nor chemotherapy had an impact on disease-free survival. In an analysis performed by Shim and Suit, eight patients with ES underwent perioperative or definitive RT [85]. Five of them were irradiated postoperatively, three due to primary tumor and two due to recurrent disease without previous RT. One patient received neoadjuvant RT. Among the aforementioned six patients, only one developed local recurrence; the 7-year risk of local control was calculated as 82% +/- 18%. The remaining two patients received definitive RT without surgery. The first patient with a 22 cm primary ES died at six months with persistent local disease and distant metastases. The second one with a small recurrent ES localized forearm achieved sustainable local control; however, this patient developed distant metastases. Nevertheless, radical surgery with conventionally fractionated perioperative RT remains a standard of care in ES [86]. Participation in clinical trials with altered fractionation and combined therapy is encouraged if available.

### 5.2. Radiotherapy of Recurrent Disease

There are no data regarding the role of RT in recurrent or metastatic ES. Perioperative RT should be considered in recurrent ES if it was not applied for the primary tumor. In the case of in-field or near-field recurrence, reirradiation could be challenging. Local recurrence with limited volume in patients who previously received external beam RT may be managed with perioperative or definitive brachytherapy [87]. Larger recurrent ES may benefit from combined treatment, namely RT with chemotherapy or RT with hyperthermia. Sixteen patients treated with moderately hypofractionated RT and hyperthermia for radiation-induced thoracic sarcomas presented good local control and acceptable toxicity for such an approach [88]. Definitive radiotherapy could be applied in selected patients with oligometastatic ES (Figure 4B–D). There is a growing number of evidence that stereotactic body radiotherapy (SBRT) is beneficial in patients with oligometastatic sarcomas, providing local control around 90% [89,90]. Palliative RT should be considered in the case of symptomatic ES metastases.

## 6. Systemic Therapy

### 6.1. Neoadjuvant and Adjuvant Therapy

In some cases, perioperative chemotherapy can be considered. Most of the analyses report the use of perioperative chemotherapy in 20.0–33.3% of patients [27,71,82,91]; however, there are reports where more than half of patients have received it [24,92]. Perioperative chemotherapy is mostly used in cases of large- sized, high -grade tumors with or without incomplete resection and/or in cases of metastasis [82,92]. In an Italian study, isolated limb perfusion (ILP) with tumor necrosis factor (TNF)-based regiments, epirubicin, cisplatin, or melphalan, was used preoperatively in 37% of patients. Other regimens included doxorubicin in monotherapy, VAIA (vincristine, doxorubicin, ifosfamide, actinomycin-D), and CyVADIC (cyclofosfamide, vincristine, doxorubicin, and dacarbazine) [27]. In the studies from Royal Marsden Hospital, Japan, and French Sarcoma Group, doxorubicin with ifosfamide was most commonly used in those settings [13,24,91,93]. Kim et al. [27] showed that there was no influence of adjuvant chemotherapy on relapse-free survival, but the majority of the studies did not report the specific outcomes in patients receiving perioperative chemotherapy; thus, it is difficult to conclude about its beneficiary role.

In pediatric patients, partial responses to neoadjuvant chemotherapy (ifosfamide and doxorubicin) with or without radiotherapy were seen in 50% of patients. In adults, responses after neoadjuvant chemotherapy alone are seen in 0–15% of patients [9,24,91]. There is no report on the association between perioperative chemotherapy and overall survival (OS), distant metastases-free survival (DMFS), or loco-regional-free survival (LRFS) [27,71,82,92]. A Japanese study has reported that chemotherapy was associated with shorter OS and DMFS; however, it was not validated in the multivariate model, and patients with more aggressive tumors were receiving chemotherapy more often [93]. However, in the retrospective analyses from Dutch centers, patients treated with curative intent have undergone ILP with TNF and melphalan, which resulted in the decrease of the tumor size and created an opportunity to perform R1 resection [23].

### 6.2. Systemic Therapy in Advanced Disease

There is no high-quality evidence on systemic therapy in advanced ES. Most of the available data come from small retrospective studies, case reports, and single patients with ES treated in clinical trials for STS therapies. The most comprehensive evidence comes from a recent multi-institutional case series performed by Frezza et al. [94], which gathered data on 115 patients with advanced or metastatic ES, who were not treated with chemotherapy in perioperative settings. The most common therapy was anthracycline-based regimens (anthracycline alone or combined with ifosfamide or other cytotoxic drugs) used in 85 patients. Anthracycline-based regimens were also the most common in other studies and used in the majority of patients (60–100% of cases) [12,13,27,31,84,91,95]. Anthracyclines are mostly combined with ifosfamide, other cytotoxic drugs such as vincristine, dacarbazine, actinomycin D, carboplatin, or cyclofosfamide, or used as a single agent [9,23,27,31,91]. The other most common drugs are gemcitabine with docetaxel used in approximately 15–40% of patients [27,94,95] and pazopanib in 10–20% of patients [27,94]. Other reported chemotherapeutic agents include high-dose ifosfamide, trofosfamide, gemcitabine with cisplatin, and cisplatin with dacarbazine [95].

Pulled data from 4 EORTC (The European Organisation for Research and Treatment of Cancer) studies (62012, 62043, 62072, 62091) [96] enable identifying 27 patients with ES out of 976 patients treated in those trials. In the first line, objective responses were observed in patients treated with pazopanib (objective response rate (ORR) 100%, 2/2), trabectedin (33.3%, 1/3), and doxorubicin with ifosfamide (12.5%, 1/8) with no responses in patients receiving doxorubicin alone. The median progression-free survival (PFS) in the first line was 4.04 months and a median OS of 10.93 months. In the second line, only pazopanib was tested with an ORR of 11.1% (1/9) and median PFS of 2.73 months. The response rate in all patients was 22.2% and seemed to be similar to pooled populations of other STS subtypes, ranging from 5% to 25% in the different trials [96]. Similar results were reported for the retrospective cohort. In patients treated in Royal Marsden Hospital, three partial responses were observed in the first line, all in patients with the classic type of ES. Of 14 patients with SD, 7 had it for over six months. The median PFS was 6.76 months (95% CI 23–35), and 3- and 6-month PFS rates were 85% and 53%, respectively. The median OS from commencing palliative chemotherapy was 12 months (95% CI 29–73). Six-month OS was 79%, and the 12-month OS rate was 46% [91]. Generally, patients who received palliative chemotherapy have significantly longer OS than those who did not receive chemotherapy (medianOS: 16.8 versus 8.7 months, *p* = 0.044) [27]. Moreover, the effect of chemotherapy was observed only in patients with the proximal type of ES [27].

Anthracycline-based therapy is associated with the best results from all available chemotherapeutics. In a study by Frezza et al. [94], the ORR to anthracycline-based regimens was 22% (1 complete response (CR), 18 partial responses (PR)), disease control rate (DCR) was 75% with a median PFS of 6 months. A higher response rate was noticed in the proximal morphological type than the classic type (26 versus 19%, *p* = 0.44); however, results were not statistically significant. There were no differences in response rates between patients treated with anthracycline in monotherapy or combination with ifosfamide [94]. Responses to doxorubicin and ifosfamide chemotherapy were also reported in children and adolescents with ES [9]. Contrary data come from a multicenter analysis performed by Pink et al. [95], who reported 0% ORR and 56% DCR; however, the population (*n* = 13) was much smaller than those in the analyses by Frezza et al. 

Gemcitabine-based regimens are the second most common. In 12 patients treated with gemcitabine-based schemes reported by Pink et al. [95], the ORR and DCR rates were 58% and 83%, respectively. The median PFS was eight months in all patients, and nine months in patients treated in a first line [95]. Worse results were reported by Frezza et al. [94]. In their analyses, the ORR was 27%, the DCR was 66%, and patients had a median PFS of 4 months. A trend toward a higher response rate was reported in the classic morphological type compared to the proximal type (30% versus 22%, *p* = 0.72) and in the distal than the proximal primary site (40% versus 14%, *p* = 0.08). They have not observed any differences in response rates between patients treated with gemcitabine in monotherapy or combination with docetaxel [94]. 

Evidence supporting the use of cytotoxic drugs other than anthracyclines and gemcitabine is weak and incidental. There is only a case report about a complete remission of pulmonary metastases on treatment with vinorelbine (17–30 mg/m2 every 2–4 weeks) with a durable response of four years [97] and a case of a patient with a partial response with the duration of treatment of 27.4 months [98].

Many expectations arose around targeted therapies for ES. Pazopanib was the first targeted agent available for the treatment of ES; however, results are not satisfactory. Frezza et al. [94] did not observe any ORRs in any of the 18 patients treated with pazopanib. While 50% of patients achieved stable disease, the PFS was only three months. There are episodic case reports of successful treatment with pazopanib in patients with metastatic ES who achieved partial responses [99,100], but we must be aware of reporting bias. 

Recently, the Food and Drug Administration (FDA) has approved tazemetostat, an EZH2 inhibitor, for the treatment of adults and pediatric patients aged 16 years and older with metastatic or locally advanced epithelioid sarcoma not eligible for radical resection. The approval has been granted based on the results of cohort 5 of the phase 2 clinical trial (NCT02601950) conducted on 62 ES patients (24 in the first-line setting and 38 already pretreated) who were treated with 800 mg of tazemetostat twice daily. The disease control rate (DCR) was 26% (95% CI 15.5–38.5); the objective response rate was 15% (95% CI, 6.9–25.8). In the study, ORR was achieved in 25% of patients in the first-line setting and only 8% for those who had prior systemic treatment [50]. The median duration of response reached an impressive 16.1 months [101]. In 2020, the results from an additional ES cohort (cohort 6) from this study were reported with an ORR of 11.4% and DCR of 50% [101]. In a pooled posthoc analysis of both cohorts, the median PFS was 3.7 months and the median OS was 18 months. In addition, in the phase I trial (NCT02601937) with tazemetostat in relapsed or refractory B-cell non-Hodgkin lymphoma and advanced solid tumor, two patients with epithelioid sarcoma have achieved stable disease and remained on treatment at more than 20 months [102]. The toxicity profile of tazemetostat is favorable, with nausea and fatigue in approximately 40% of patients and grade ≥3 treatment-related events in up to 16% of patients [50,101]. Currently, tazemetostat is tested in combination with doxorubicin in Phase 1b/3 trial as a front-line therapy for patients with advanced epithelioid sarcoma [103]. 

There are also some data on the activity of dasatinib in ES. In an open-label, single-arm SARC0009 trial, 2 out of 7 patients achieved objective tumor responses according to Choi criteria with a median PFS of 7.9 months and a 6-month PFS rate of 57%. However, OS was poor, with a 2-year OS rate of only 21% [104]. There is a case of long-term disease stabilization (>32 months) on sunitinib in the 3^rd^ line, after progression on two lines of chemotherapy [105].

Clinical trials of immunotherapy in STS, including ES, are currently ongoing. So far, there are available data on one patient with ES who achieved PR in the KEYNOTE-051 study with pembrolizumab in pediatric patients with PD-L1-positive (programmed death-ligand 1), advanced, relapsed, or refractory solid tumor [106]. In a study with nivolumab, one case of partial response was observed after four cycles in a 24-year-old man with a proximal-type ES metastatic to the lung; however, the patient progressed after four additional cycles. The second patient with ES had disease progression at the first evaluation [107].

The systemic therapy of ES remains challenging and is not supported by high-quality evidence. In the light of available data, anthracycline-based regimens, preferably in combination with ifosfamide, are preferred as a first-line treatment of patients with advanced or metastatic ES (Table 1). After the approval of tazemetostat, the treatment strategy for palliative ES may change. Tazemetostat was the most effective in the first line, and if it becomes also registered outside the USA and widely accessible on the market, it can replace anthracyclines. Differences in response to chemotherapy are observed between proximal and distal types, which also influence the choice of therapy. Based on the retrospective analyses, anthracyclines seems to be more effective in proximal, while gemcitabine-based regimens are more effective in the distal ES type. Nevertheless, those regimens were never compared head-to-head in the clinical trial; thus, those observations should be interpreted with caution. The available data are obscure and limited, which did not help to stratify patients based on the treatment outcomes. 

Other options include gemcitabine-based chemotherapy and tazemetostat. In the light of discrepant reports on response rates to gemcitabine-based regimens and lack of head-to-head in a clinical trial, the selection was that appropriate therapy should be individualized, discussed in a multidisciplinary team and led by an experienced center. There is no data about the optimal therapy scheme for second and further lines. Practice changing results of currently ongoing clinical trials are highly awaited (Table 2).

## 7. ES in Children

In children, ES is classified in the heterogeneous group of non-rhabdomyosarcoma soft-tissue sarcomas (NRSTS). It accounts for about 5% of pediatric NRSTS cases [9,108]. Only a few data on clinical features and optimal treatment of pediatric patients are described in the literature. ES is most common in older children (7–14 years) with a slight male predominance, but newborn cases have also been reported [9,10,109,110,111]. A typical location for pediatric ES are extremities, especially arms and hands. There are some characteristics, such as location in superficial distal sites, indolent growth rate, and the tendency to locoregional relapses [9,10,110,111]. 

The risk stratification system for young patients with NRSTS divided patients into three prognostic groups: low, intermediate, and high risk, with significantly different event-free survival (EFS) and OS (88.9%, 65%, 21.2% and 96.2%, 79.2%, 35.5%, respectively). Five factors were used in this system: Pediatric Oncology Group (POG) grade, tumor size, metastatic status, the extent of resection, and marginal status [108].

Treatment strategies in ES have not changed significantly over the years. The cornerstone of the treatment of localized disease is wide surgical resection. However, multimodal therapy, including chemotherapy and irradiation, is recommended for most children. Only adequately excised small tumors can be safely treated with surgery alone [9,109,110]. 

ES is reported to be chemosensitive, and the response rate to anthracycline-based regimes is about 22% [9]. Chemotherapy includes vincristine and dactinomycin in combination with an alkylating agent and anthracycline, etoposide, or carboplatin depending on the patient’s risk group. Infants <12 months should receive reduced doses of chemotherapy [110].

Radiotherapy is given to patients who are at risk of local recurrence. The European pediatric Soft Tissue Sarcoma Group (EpSSG) protocol recommends radiation therapy doses in the range of 50.4–59.4 Gy depending on resection time and surgical margins: 50.4 Gy preoperatively and after R0 resection, 54 Gy after R1 resection, and 59.4 Gy for gross tumor mass. The dose of radiation for metastases is determined individually depending on many factors, such as the patient’s age, location, and the number of metastases [109].

Radiotherapy before surgery has several advantages, which are especially important in children: lower total dose and lower irradiation volume, greater efficacy in non-hypoxic tissues after resection, and reduced risk of secondary neoplasia by removing most of the irradiated tissue. The overall results for children and adolescents with ES are comparable with those in patients with other NRSTS [112]. Pediatric patients appear to have a more favorable prognosis compared to adults because they are more likely to be diagnosed with distal-type ES and are less likely to have nodal or metastatic involvement at diagnosis. A recent report from the Italian Soft Tissue Sarcoma Committee demonstrated 5-year EFS and OS in the pediatric group 61.7% and 92.4%, respectively [9]. Sparber–Sauer et al. did not confirm these results. In the whole evaluated group of 67 patients, they reported EFS and OS of only 29% and 48%, respectively (35% and 58% for patients with localized disease). Patients with metastatic disease had a 5-year EFS rate of 7% and an OS rate of 9% [110]. There is very little data on relapses in younger patients, and optimal therapy is still undefined. More research is needed to assess the benefits of new targeted therapies.

## 8. Prognosis

The survival of patients with ES is unsatisfactory. Depending on the study, the 5-year overall survival rates from 25% to 70% [12,13,24,69,70,74,93]. Five years on from primary tumor diagnosis, metastatic spread is found in 30% to 75% of patients [70,72,74]. In the analysis of the Surveillance, Epidemiology, and End Results (SEER) Database, 998 ES cases were identified. The reported 5-year disease-specific survival was 55.7%, the 10-year OS rate was 60.4%, the recurrence rate was 63.4%, and the metastasis rate was 40.3%. While patients diagnosed after the year 2000 had a worse prognosis, the results might have been biased by the selection or underreporting of ES [22]. 

Several prognostic and predictive factors were reported. Patients older than 55 years are to be at a higher risk of death [22]; however, age prognostic significance in ES is still under debate [13,74]. The larger size of the ES primary tumor at primary diagnosis is correlated with shorter overall survival [22]. Tumor size >5 cm is an independent negative predictor of outcome [13,22]; however, some reports do not confirm this observation [74]. High-grade tumors (grades 3 and 4) are correlated with worse outcomes, including shorter overall survival than grade 2, but in a majority of studies, there was no association with risk of recurrence [22,24,74,113]. Tumor necrosis over 50%, the presence of vascular invasion, and a high mitotic rate ≥20/10 HPF (high power field) are significantly correlated with poor outcomes [19,60]. In some reports, no association of ES survival with histological subtypes was found [60]. Nevertheless, the proximal subtype is most often associated with a poorer prognosis than the distant subtype of ES [22,60,72,113], even though no differences in the recurrence rate were reported [74]. The nodular pattern is characterized by an increased risk of local recurrence [71]; however, without a significant impact on survival [12]. Finally, deep axial location compared to superficial axial or appendicular is associated with poorer prognosis and a higher recurrence rate [24,60,74]. The presence of lymph nodes metastases at the primary diagnosis and the presence of distant metastases is significantly correlated with the poor prognosis [74,114]. The diagnosis of metastatic disease doubles the risk of death [22].

## 9. Conclusions

ES cells harbor mesenchymal and epithelioid features. ES cells express vimentin, cytokeratins, and epithelial membrane antigen (EMA). Loss of integrase interactor 1 (INI1) protein function is the most common alteration found in ES, which is reported in almost 90% of patients [37]. The survival of patients with ES is unsatisfactory. Five-year disease-specific survival is about 55%. The treatment of choice of ES is a radical excision with microscopically radical margins and perioperative radiotherapy. As compared to other types of soft tissue sarcomas, the spread to regional lymph nodes can occur more frequently (>20%) Anthracycline-based chemoregimens are associated with highest the ORR of 22% [94]. Recently, the FDA has approved tazemetostat, an EZH2 methyltransferase, for the treatment of adults and pediatric patients aged 16 years and older with metastatic or locally advanced epithelioid sarcoma not eligible for radical resection [50].

## Figures and Tables

**Table 1:** Systemic therapy regimens available for epithelioid sarcoma treatment.

Drugs	Dose	Frequency	ORR	Ref.
Doxorubicin	75 mg/m^2^	d. 1 every 3 weeks (bolus or 72h infusion)	NA 0% (0/14) *	[91] [96]
Doxorubicin Ifosfamide	20 mg/m^2^ 3 g/m^2^	d. 1–3 every 3 weeks d. 1–3 every 3 weeks	NA	[91]
Doxorubicin Ifosfamide	20 mg/m^2^ 2 g/m^2^	d. 1–3 every 3 weeks d. 1–3 every 3 weeks	NA	[31]
Doxorubicin Ifosfamide (perioperative, 3 cycles)	20 mg/m^2^ 1.5 mg/m^2^	d. 1–3 every 3 weeks d. 1–4 every 3 weeks	NA	[92]
Doxorubicin Ifosfamide	25 mg/m^2^ 2,5 mg/m^2^	d. 1–3 every 3 weeks d. 1–4 every 3 weeks	12.5% (1/8) *	[96]
Anthracycline-based regimens	NA	NA	19/85 (22%) ^#^	[94]
Trabectedin	1.5 mg/m^2^	Every 3 weeks	NA	[91]
Ifosfamide Vincristine Doxorubicin (IVAD)	3 g/m^2^ 1.4 mg/m^2^ 20 mg/m^2^	d. 1–3 every 3 weeks d. 1 every 3 weeks d. 1–3 every 3 weeks	NA	[91]
Pazopanib	800 mg	daily	0% (0/18) ^#^ 100% (2/2) * 100% (2/2) ^ 100% (1/1) ^	[94] [96] [99] [100]
Trabectedin	1.5 mg/m^2^	d. 1 every 3 weeks (24h infusion)	33.3% (1/3) *	[96]
Gemcitabine Docetaxel	900 mg/m^2^ 100 mg/m^2^	d. 1, 8 every 3 weeks d. 8 every 3 weeks	58% (7/12) ^#^	[95]
Gemcitabine-based	NA	NA	27% (11/41) ^#^	[94]
Vinorelbin	17–30 mg/m^2^	d. 1 every 2–4 weeks	100% (1/1) ^ 100% (1/1) ^	[97] [97,98]
Tazemetostat	800 mg	bidaily	15% (9/62) *	[50]
Dasatinib	70 mg	bidaily	28.6% (2/7)	[104]
Nivolumab	3 mg/kg bw	every 2 weeks	50% (1/2) ^#^	[107]
Pembrolizumab	2 mg/kg bw	every 3 weeks	100% (1.1) *	[106]

*—randomized clinical trials, ^—case reports, ^#^—retrospective study, Bw—body weight, d—day, NA—not available, ORR—objective response rates.

**Table 2:** Ongoing clinical trials in epithelioid sarcoma.

Number	Drug	Status	Details	Additional Information
NCT04204941	Doxorubicin + Tazemetostat/placebo	Recruiting	Tazemetostat: 400/600/800 mg BID Doxorubicin: 75 m^2^ d. 1 every 3 weeks for 6 cycles	Phase 1b/3
NCT03009201	Ribociclib + doxorubicin	Active, not recruiting	Ribociclib d. 1-6 400 mg and Doxorubicin 75 mg/m^2^ d. 10, every 3 weeks for 6 cycles, than ribocyclib daily	
NCT01532687	Gemcitabine + pazopanib/placebo	Active, not recruiting	NA	
NCT01154452	Vismodegib + Gamma–Secretase/Notch Signaling Pathway Inhibitor RO4929097 or placebo	Completed	Vismodegib 150 mg QD and RO4929097 15 mg QD	No specific results for epithelioid sarcoma available

BID—bidaily, d—daily, QD—quaterdaily.
